# Note on Anti-Toxine

**Published:** 1896-05

**Authors:** 


### NOTE ON ANTI-TOXINE.

A profound sensation has been created among medical men of Germany and France through the fatal effect attending the administration of anti-diphtheria serum to the child of an eminent Berlin physician. A servant in the physician's household, showing signs of diphtheria, the doctor, to prevent his child from contracting the disease, administered an injection of the serum, and the child died in a few minutes.

United States Commercial Agent Moore, at Weimar, has made the incident the subject of a special report to the State Department, in which, after telling of the theories put forth by the medical authorities to account for the fatal action of the serum, he declares the child died from nervous shock, although it has been decided that the injection of the serum into a healthy person for prevention is a dangerous practice.—*Philadelphia Evening Bulletin*, May 18th, 1896.

